# Cardiac serum marker alterations after intraoperative radiotherapy with low-energy x-rays in early breast cancer as an indicator of possible cardiac toxicity

**DOI:** 10.1007/s00066-020-01671-3

**Published:** 2020-08-19

**Authors:** Stefan Stefanovic, Sebastian Berlit, Elena Sperk, Frederik Wenz, Christel Weiß, Frederik Trinkmann, Marc Sütterlin, Benjamin Tuschy

**Affiliations:** 1 Department of Gynecology and Obstetrics, University Medical Center Mannheim, Heidelberg University, Theodor-Kutzer-Ufer 1–3, 68167 Mannheim, Germany; 2 Department of Radiation Oncology, University Medical Center Mannheim, Heidelberg University, Mannheim, Germany; 3 Department of Medical Statistics and Biomathematics, Medical Faculty Mannheim, Heidelberg University, Mannheim, Germany; 4 First Department of Medicine (Cardiology, Angiology, Pulmonary and Intensive Care), University Medical, Center Mannheim, Heidelberg University, Mannheim, Germany

**Keywords:** Tumor bed boost, Intrabeam, Heart enzymes, Breast-conserving surgery, Early onset Cardiac damage, Cardiotoxicity

## Abstract

**Purpose:**

To assess acute cardiac toxicity caused by intraoperative radiotherapy (IORT) with low-energy x‑rays for early breast cancer.

**Methods:**

We prospectively analyzed pre- and postoperative troponin I and NT-proBNP in 94 women who underwent breast-conserving surgery between 2013 and 2017 at the Department of Gynecology and Obstetrics of the University Medical Center Mannheim, Germany. Thirty-nine women received IORT using low-energy x‑rays during breast-conserving surgery while 55 patients without IORT formed the control group. Demographic and surgical parameters as well as cardiac markers were evaluated.

**Results:**

There were no significant differences concerning age and side of breast cancer between the groups. Furthermore, no significant difference between the troponin I assays of the IORT and control groups could be found (preoperatively: 0.017 ± 0.006 ng/ml vs. 0.018 ± 0.008 ng/ml; *p* = 0.5105; postoperatively: 0.019 ± 0.012 ng/ml vs. 0.018 ± 0.010 ng/ml; *p* = 0.6225). N‑terminal fragment of B‑type natriuretic peptide (NT-proBNP) was significantly higher in the control group 24 h after surgery (preoperatively: 158.154 ± 169.427 pg/ml vs. 162.109 ± 147.343 pg/ml; *p* = 0.56; postoperatively: 168.846 ± 160.227 pg/ml vs. 232.527 ± 188.957 pg/ml; *p* = 0.0279).

**Conclusion:**

Troponin I levels as a marker of acute cardiac toxicity did not show any significant differences in patients who received IORT during breast-conserving surgery compared to those who did not.

**Electronic supplementary material:**

The online version of this article (10.1007/s00066-020-01671-3) contains supplementary material, which is available to authorized users.

## Introduction

With a lifetime risk of almost 10%, breast cancer is the most common malignant tumor in the female population worldwide. Early breast cancer treatment in the majority of cases consists of breast-conserving surgery (BCS), which is typically combined with axillary sentinel lymph node biopsy (SNB) followed by external beam whole-breast radiotherapy (EBRT). Other crucial therapeutic principles in the treatment of breast cancer are chemotherapy, endocrine, and targeted therapies.

Over recent years radiotherapy techniques have improved in terms of sophistication and versatility and a decrease in the risk of local relapse was attained by applying an additional tumor bed boost of 10–20 Gy in high-risk patients [1]. A problem of the external application of the tumor bed boost application lies in the potential risk of missing the target, which is considered to be 20–90% [1]. This risk of missing the tumor bed can be reduced by the application of intraoperative radiotherapy (IORT) to the tumor bed during BCS immediately after removal of the tumor [2]. Besides minimizing the risk of “geographical miss,” IORT can shorten the interval between tumor excision and the beginning of adjuvant radiotherapy, thereby making a “temporal miss” unlikely as well [2].

Depending on the technique used as well as the indication, IORT can be applied as a tumor bed boost followed by EBRT (in high-risk patients) or as a standalone therapeutic modality in the sense of accelerated partial breast irradiation (in low-risk patients) [3–5].

The mobile Intrabeam® (Carl Zeiss Surgical, Oberkochen, Germany) device is a miniature x‑ray source with a maximum of 50 kV which has been used for IORT during BCS in selected patients at the University Medical Center Mannheim since February 2002 [5–8]. Thus far it seems that in early low-risk breast cancer settings IORT is non-inferior to EBRT when it comes to local cancer recurrence rates and breast cancer-related mortality [5, 9]. However, in the TARGIT‑A trial, non-inferiority was only shown in the pre-pathology cohort and patients received additional EBRT as indicated [9]. Also, IORT using Intrabeam has been reported to deliver significantly less radiation to normal tissues when compared to EBRT [5–7, 10, 11]. Woolf et al. demonstrated that the radiation exposure (using gamma-H2AX in circulating lymphocytes as a biological marker of radiation dose) to the intrathoracic organs (including heart and intrathoracic great vessels) is significantly lower with IORT compared to EBRT [10, 11]. Moreover, in a previous study by our group, postoperative complications were demonstrated to be rare and immediate toxicity was low [12].

### Adverse cardiac effects

Cardiac toxicity is one of the most feared complications associated with radiotherapy. Subclinical cardiac damage occurs in >50% of breast cancer survivors treated with radiation therapy [13]. It is known to be mostly a late-onset adverse effect of radiotherapy and might clinically manifest even decades after the original treatment. Its significance is caused foremost by its irreversibility and the quality of life forfeit [14]. Exposure of the heart to ionizing radiation during radiotherapy for breast cancer increases the subsequent rate of ischemic heart disease. The increase is proportional to the mean dose to the heart. Previous studies have shown that the rate of major coronary events increases by 7.4% for each elevated Gy in the mean radiation dose delivered to the heart [15].

In left-sided breast cancer patients EBRT has been shown to raise cardiovascular mortality and morbidity compared to right-sided breast cancer patients [14].

Cardiotoxicity risk is increased by the following individual risk factors: age, smoking, hypercholesterolemia, weight, hypertension, diabetes, family history, and additional oncologic treatments such as targeted, endocrine, and chemotherapies. Hooning et al. showed a significant negative correlation between cardiopathy risk and age at radiotherapy in breast cancer patients [16]. Patients below 35 years of age seem to be especially vulnerable to radiation-induced cardiopathy [16].

Cardiotoxicity after IORT is assumed to be lower than after EBRT, as IORT delivers the lowest maximum dose to the heart in comparison to EBRT [17].

### Cardiac markers

Cardiac markers play an important role not only in the diagnosis and assessment of cardiac diseases, but also concerning decision-making and risk stratification. Cardiac troponin T (cTnT) and troponin I (cTnI) are cardiac regulatory proteins that control the calcium-mediated interaction between actin and myosin. While cardiac troponin T is expressed to a small extent in skeletal muscle, cTnI has not been identified outside the myocardium [18]. Cardiac troponins are established biomarkers of acute myocardial injury with very high sensitivity, and are independently predictive of adverse outcomes following noncardiac surgery [19].

A previous study of Skyttä et al. demonstrated that high-sensitivity cardiac troponin T (hscTnT) levels increased during whole-breast adjuvant radiotherapy (RT) in every fifth patient. Moreover, the increase in hscTnT release was positively associated with cardiac radiation doses and with minor changes in left ventricle diastolic function, suggesting that RT caused subclinical myocardial damage [20].

B‑type natriuretic peptide (BNP) is a biomarker that is synthesized and secreted in ventricular myocytes and, to a lesser extent, in the atrial myocardium [19]. It is synthesized as a 108-amino acid prohormone (proBNP) which is cleaved to the 32-residue BNP and the 76-residue N‑terminal fragment of proBNP (NT-proBNP) [21]. The main stimulus for the release of BNP is increased myocardial wall stretch mediated by pressure or volume loading [19].

BNP protects the heart from adverse consequences of overload by increasing natriuresis and diuresis, relaxing vascular smooth muscle, inhibiting the renin–angiotensin–aldosterone system, and by counteracting cardiac hypertrophy and fibrosis [21]. It plays an important role in the diagnosis of congestive heart failure, severity stratification, and therapy control.

In a previous study, patients with left-sided breast cancer showed higher values of NT-proBNP 5 to 22 months after RT when compared with non-RT-treated matched patients, increasing in correlation with high doses in small volumes of heart and ventricle [22].

Jingu et al. could show higher BNP concentrations after radiotherapy of esophageal cancer [23]. Nellesson et al. found an increase in both troponin I and BNP levels in their weekly serum controls during RT in 23 patients receiving RT because of either breast or lung cancer [24].

Hence, BNP concentration might be an early indicator of radiation-induced myocardial damage [24].

The aim of this study was to assess acute cardiotoxicity caused by IORT with low-energy x‑rays using the biomarkers NT-proBNP and troponin I as indicators.

## Materials and methods

This study was designed as monocentric and prospective proof of principle study. Eligible for enrollment in the study were all women undergoing breast-conserving surgery who had confirmed invasive breast cancer and were capable of informed consent.

The primary endpoint of the study was to prove that cardiac impact within IORT as an integral part of breast-conserving surgery does not trigger acute cardiac toxicity. To this end, the heart parameters troponin I and NT-proBNP were measured prior to and after breast-conserving surgery in patients with and without IORT.

The eligibility criteria for IORT are multimodal. The decisive criteria included tumor size, tumor–skin distance, intraoperative situs, and patient’s motivation/consent for IORT.

Between 2013 and 2017, a total of 94 women with early breast cancer were included in this study. The local ethics committee approved the protocol and all participants signed informed consent prior to study enrollment. Thirty-nine of the included patients were treated by IORT using the mobile Intrabeam® device (Carl Zeiss Surgical, Oberkochen, Germany) during BCS at the University Medical Center Mannheim, Germany. This system consists of a miniaturized linear accelerator emitting low-energy x‑ray photons (maximum 50 kV), a floor stand and a control unit. The control group consisted of 55 women who did not receive IORT during BCS. The surgical and radiotherapeutic procedures were performed in accordance with hospital and national protocols.

Troponin I and NT-proBNP levels were measured before and 24 h after surgery in all patients. All data were collected in an Excel™ (Microsoft Corporation, Redmond, Seattle, WA, USA) datasheet. After a thorough check for faulty entries, the data were transferred for statistical analysis. All computations were performed using the SAS®, version 9.4, statistics software (SAS Institute, Cary, NC, USA).

Pre- and post-surgery leukocyte counts, hemoglobin, troponin I, and NT-proBNP values were compared using a Mann–Whitney *U*-test. The same test was used in an attempt to detect between-group differences in the absolute changes (from before surgery to 24 h after surgery) in leukocyte counts and hemoglobin, troponin I and NT-proBNP values. The chi-square test or Fisher’s test was performed to compare the groups in terms of breast cancer characteristics (not including exact tumor size) and location and previous history of cardiovascular diseases. The mean patient age, tumor size, and surgery duration were compared using a t-test.

## Results

The data of interest to the investigators were available for all the enrolled patients except for the missing postoperative leukocyte count and hemoglobin level datapoints for 5 patients in the non-IORT group.

There were no significant differences in age (*p* = 0.4579), overall cardiovascular disease history (*p* = 0.8901), smoking status (*p* = 0.5068), and axillary surgical management (*p* = 0.8732) between the two subgroups as shown in Table 1. Statistically nonsignificant differences were observed in the frequency of antecedent cardiovascular disease barring arterial hypertension (10 patients in the non-IORT vs. 1 in the IORT group; *p* = 0.075). The location of the breast cancer lesions was comparable in women with and without IORT (Table 2) while the duration of surgery was significantly longer (*p* < 0.0001) in the IORT group (Table 1). Two patients from the IORT group received neoadjuvant systemic therapy with epirubicin and cyclophosphamide followed by a taxane, and one patient from the control group underwent neoadjuvant treatment with epirubicin and cyclophosphamide followed by paclitaxel enhanced by dual HER2-blockade with trastuzumab and pertuzumab (Table 2).

**Table 1:** Demographic and surgical parameters of the study (IORT) and control collective (non-IORT; *N* = 94)

Variable	*With IORT* (*n* = 39) mean ± SD respective frequencies	*Without IORT* (*n* = 55) mean ± SD respective frequencies	*p*-value
*Age (years)*	59.7 ± 11.5	61.4 ± 10.5	0.4579
*Chronic diseases*			
Hypertonia	18 (46.2%)	25 (45.5%)	0.95
Myocardial infarction	0 (0%)	4 (7.3%)	0.139
Cardiomyopathy	0 (0%)	1 (1.8%)	1
Cardiac arrhythmia	1 (2.6%)	3 (5.5%)	0.64
Coronary heart disease	0 (0%)	2 (3.6%)	0.51
*Smoking status*	7 (18.0%)	13 (23.6%)	0.5068
*Duration of surgery (min)*	142.5 ± 30.1	93.2 ± 37.9	<0.0001
*Sentinel node biopsy procedure*	31 (79.5%)	44 (80%)	0.8732
Positive	3 (7.7%)	6 (10.9%)	
Negative	28 (71.8%)	38 (69.1%)	

*SD* standard deviation, *IORT* intraoperative radiotherapy

**Table 2:** Breast cancer-related parameters of the study (IORT) and control collective (non-IORT)

Variable	*With IORT* (*n* = 39) frequency (%)	*Without IORT* (*n* = 55) frequency (%)	*p*-value
*Localization of breast cancer/surgery*			0.4304
Left	28 (71.8)	33 (60)	
Right	11 (28.2)	21 (38.2)	
Both sides	0 (0)	1 (1.8)	
*TNM status*			0.0117
ypT0	2 (5.1)	1 (1.8)	
pT1a	6 (15.4)	3 (5.5)	
pT1b	6 (15.4)	4 (7.3)	
pT1c	17 (43.6)	28 (50.9)	
pT2	8 (20.5)	17 (30.9)	
pT3	0 (0)	1 (1.8)	
pT4	0 (0)	1 (1.8)	
pN0	31 (79.5)	42 (76.4)	1.000
pN1	8 (20.5)	12 (21.8)	
pN2	0 (0)	1 (1.8)	
cM0	20 (51.3)	29 (52.7)	1.000
M1	1 (2.6)	1 (1.8)	
Mx	18 (46.2)	25 (45.5)	
*ER positive*	32 (82.1)	50 (90.9)	0.2259
*PR positive*	30 (76.9)	46 (83.6)	0.4151
*Her2 positive*	4 (10.3)	7 (12.7)	1.000
*Neoadjuvant chemotherapy*	2 (5.1)	1 (1.8)	n.a.

*ER* estrogen receptor, *PR* progesterone receptor,* IORT* intraoperative radiotherapy

There were no differences concerning the incidence of multicentric breast cancer upon comparing the two groups (IORT *n* = 3 [7.7%] vs. control *n* = 6 [10.9%]; *p* = 0.7310), whereas tumor size showed significant differences (*p* = 0.0117) as depicted in Table 2. Furthermore, both groups lacked significant differences in terms of hormonal immunohistochemical characteristics, as depicted in Table 2.

Troponin I levels were not significantly different between the groups in the preoperative (*p* = 0.5105) or the postoperative (*p* = 0.6225) setting, as displayed in Table 3. Also, no differences were observed in the troponin I value dynamics between the groups (*p* = 0.2882). The same is true when comparing the left-sided and right-sided breast cancer patients (Table 3). There was a significant difference in the postoperative NT-proBNP levels, with a significantly lower postoperative NT-proBNP in the IORT group (168.846 ± 160.227 in the IORT-group vs. 232.527 ± 188.957 in the control group; *p* = 0.0279). Similarly, a significant difference in the absolute change in the NT-proBNP levels (from pre-surgery to post-surgery) was observed between the groups (10.692 ± 67.130 in the IORT group vs. 70.418 ± 126.080 in the control group; *p* = 0.0044). A subgroup analysis regarding laterality of the breast cancer showed significant NT-proBNP changes only among left-sided IORT and control group patients (*p* = 0.0365; Table 3).

**Table 3:** Dynamics of cardiac and other major laboratory parameters of the study and control collective before and after breast conserving surgery with/without IORT

Variable	*With IORT* (*n* = 39) mean ± SD	*Without IORT* (*n* = 55) mean ± SD	*p*-value	*With IORT left* (*n* = 28) mean ± SD	*Without IORT left* (*n* = 33) mean ± SD	*p*-value	*With IORT right* (*n* = 11) mean ± SD	*Without IORT right* (*n* = 21) mean ± SD	*p*-value
*Troponin I (ng/ml)*									
Preoperative	0.0174 ± 0.0063	0.0175 ± 0.0080	0.5105	0.0169 ± 0.0041	0.0165 ± 0.0024	0.7603	0.0185 ± 0.0102	0.0190 ± 0.0126	0.5999
Postoperative	0.0189 ± 0.0119	0.0183 ± 0.0101	0.6225	0.0193 ± 0.0132	0.0166 ± 0.0025	0.9064	0.0178 ± 0.0078	0.0210 ± 0.0159	0.4067
Delta	0.0015 ± 0.0112	0.0008 ± 0.0049	0.2882	0.0024 ± 0.0131	0.0000 ± 0.0004	1.0000	−0.0007 ± 0.0024	0.0021 ± 0.0078	0.1167
*NT-proBNP (pg/ml)*									
Preoperative	158.2 ± 169.4	162.1 ± 147.3	0.5597	154.3 ± 165.0	141.0 ± 123.5	0.9596	168.1 ± 188.3	188.2 ± 178.0	0.4628
Postoperative	168.8 ± 160.2	232.5 ± 189.0	0.0279	166.5 ± 155.9	210.0 ± 175.3	0.1458	174.8 ± 178.7	262.8 ± 211.8	0.1316
Delta	10.7 ± 67.1	70.4 ± 126.1	0.0044	12.3 ± 75.3	69.0 ± 121.4	0.0365	6.7 ± 42.6	74.6 ± 138.7	0.0808
*Hemoglobin levels (g/dl)*									
Preoperative	13.5 ± 1.3	13.3 ± 1.1	0.6286	13.5 ± 1.5	13.5 ± 1.0	0.5192	13.5 ± 0.9	13.0 ± 1.3	0.1603
Postoperative	12.7 ± 1.2	12.8 ± 1.3	0.4080	12.6 ± 1.3	12.9 ± 1.1	0.3348	12.8 ± 1.0	12.7 ± 1.6	0.9105
Delta	−0.8 ± 1.0	−0.5 ± 0.7	0.2500	−0.8 ± 1.1	−0.6 ± 0.7	0.8792	−0.7 ± 0.7	−0.2 ± 0.7	0.0754
*Leukocytes (per nl)*									
Preoperative	7.5 ± 2.2	7.4 ± 2.0	0.8780	7.8 ± 2.1	7.5 ± 1.7	0.6074	6.9 ± 2.2	7.2 ± 2.2	0.7062
Postoperative	11.4 ± 2.6	11.4 ± 3.2	0.8201	11.6 ± 2.5	11.5 ± 2.9	0.6111	10.9 ± 2.8	11.1 ± 3.7	0.8750
Delta	3.9 ± 2.0	3.9 ± 2.9	0.9901	3.8 ± 2.2	4.0 ± 2.6	0.9214	4.1 ± 1.6	3.7 ± 3.5	0.9821

*SD* standard deviation,* IORT* intraoperative radiotherapy

However, preoperative NT-proBNP levels were similar (158.154 ± 169.427 in the IORT group vs. 162.109 ± 147.343 in the control group; *p* = 0.5597; Table 3).

The WBC counts as well as the hemoglobin levels and their dynamics (pre- versus postoperative) showed no significant discrepancies between groups (Table 3).

**Fig. 1:**
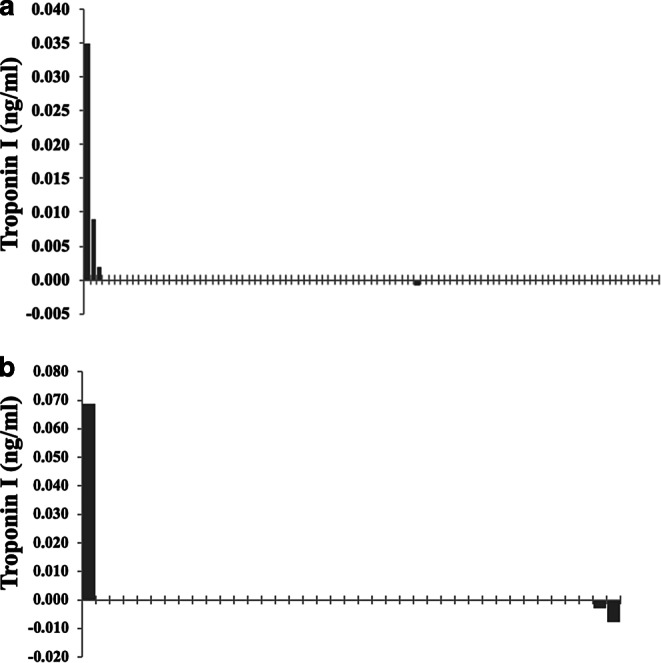
Waterfall plots representing the individual changes in troponin I levels (ng/ml), each plot on the x-axis representing a singular patient. **a** Control group, **b** intraoperative radiotherapy group

**Fig. 2:**
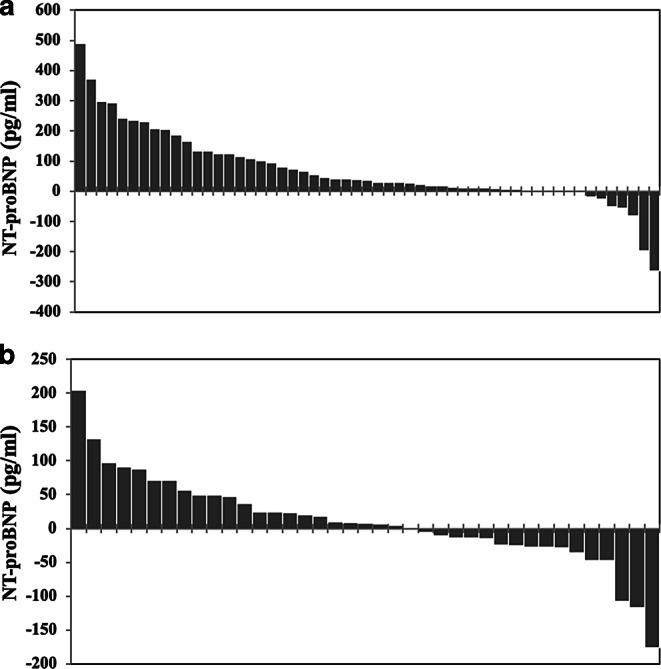
Waterfall plots representing the individual changes in NT-proBNP levels (pg/ml), each plot on the x-axis representing a singular patient. **a** Control group, **b** intraoperative radiotherapy group

**Fig. 3:**
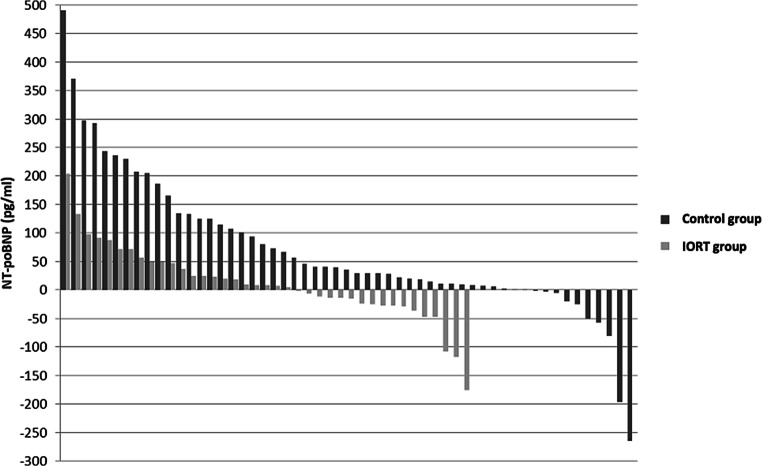
NT-proBNP (pg/ml) dynamics in the intraoperative radiotherapy (*IORT*) group and the control group, each plot on the x-axis representing a singular patient

**Fig. 4:**
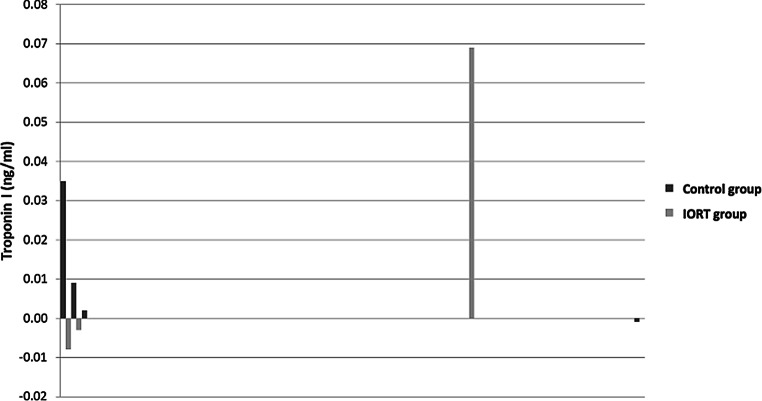
Troponin I (ng/ml) dynamics in the intraoperative radiotherapy (*IORT*) group and the control group, each plot on the x-axis representing a singular patient

For the purpose of better visualization, waterfall plots depicting the dynamics of cardiac markers of every individual patient were included (Figs. 1 and 2), as well as a comparison of the dynamics of the cardiac markers depicting the IORT group alongside the control group (Figs. 3 and 4).

## Discussion

Postoperative radiotherapy is standard of care for patients with early breast cancer and has led to a decrease in cancer recurrence and mortality rates [25]. However, it is not without risks and its unintentional cardiotoxic effects have been observed time and again [26]. The effect of radiation on the heart predictably depends on the percentage of the heart volume that is irradiated and on the cumulative dose of radiation [15, 27]. The leading theory is that myocardial damage is actually a consequence of the indirect effects of a compromised cardiac vasculature [28].

Darby et al. elegantly demonstrated that there is a direct linear correlation between the mean radiation dose and the percentage increase in the rate of major coronary events in the years following EBRT [15]. The cumulative risk of cardiac death rises nonlinearly in the course of time, but the highest percentage of major coronary events was recorded during the first 10 years after radiotherapy.

The distance between the heart and the radiation source as well as the characteristics of the source itself determine the percentage of the myocardium affected by the radiation [17, 29]. As Aziz et al. have demonstrated, the Intrabeam® device emits low-energy x‑rays and has the most favorable penetration profile when compared to EBRT and MammoSite balloon brachytherapy (Proxima Therapeutics, Alpharetta, GA, USA) [17]. However, long-term cardiac toxicity remains to be studied and EBRT is added to the IORT protocol in some high-risk patients [9].

Considering all the facts stated above, one would expect the IORT technique to be significantly less cardiotoxic than those previously employed in the early breast cancer setting. This could possibly also contribute to the circumstance demonstrated by Vaidya et al., who observed a significant increase in non-cancer-related deaths in the EBRT group of the TARGIT trial as compared to the IORT group [5]. Of equal importance is the fact that the local recurrence rates were similar between the groups if there was no delay between surgery and IORT and if patients with high-risk pathologic features received EBRT after IORT.

Although the chronic effects of radiation on the heart are well known, there are a limited number of studies analyzing acute heart damage in patients undergoing EBRT, and the findings of these are ambiguous [28, 30–35]. To our knowledge, there has only been a single study assessing IORT in this context [28]. The patients were treated for early breast cancer with the Intrabeam IORT technique. However, in contrast to our enrollment criteria, Saibene et al. excluded patients with preexisting heart disease. Also, no baseline BNP measurements were performed. Nonetheless, the authors conclude that there is no evidence of acute myocardial damage in patients receiving IORT. Finally, it is difficult to compare the cardiotoxic effect of EBRT and IORT in light of this paucity of published data.

Our study also aims to assess acute cardiotoxicity of IORT with low-energy x‑rays in patients with early breast cancer using serial troponin I and NT-proBNP measurements as indicators of radiation-induced acute heart damage. The demographic and surgical parameters of the interventional and the control cohort were observed to be similar, with significant differences only in terms of surgery duration and tumor size (Table 1 and 2). As expected, the patients undergoing IORT within their breast-conserving surgery had spent more time in the operating room (OR) due to the time-consuming intraoperative radiotherapy (*p* < 0.0001). The smaller tumor size in the IORT group (*p* = 0.0117) is not surprising and is the consequence of the technical limitations of the Intrabeam® device, which has a maximum applicator size of 5 cm.

Leukocyte counts and hemoglobin concentrations did not differ significantly between the groups, demonstrating the low acute bone marrow suppression potential of IORT. Also, the prolonged surgery in the IORT group was obviously not associated with significant blood loss.

We showed that in our cohort, IORT did not influence pre- or postoperative troponin I levels. Surprisingly, while NT-proBNP levels were similar between the groups before surgery, they were significantly higher in the non-IORT group after surgery. Interestingly, in a further subgroup analysis, this difference turned out to be significant only in the left-sided breast cancer cases, as presented in Table 3. The NT-proBNP statistics are obviously not caused by a single patient’s levels, but a versatile diapason of values, as depicted in Fig. 2. The difference could conceivably be a consequence of the fact that patients with non-hypertensive cardiovascular disease were evidently overrepresented in the non-IORT group. Smoking status and mean age did not differ significantly between the groups and patients in both groups had a similar proportion of left-sided cancers. On the other hand, surgery supplemented by IORT was longer, yet it did not seem to influence NT-proBNP levels negatively.

Our results speak in favor of IORT’s acute cardiac safety and are concordant with the recent findings of Saibene et al. in terms of the absence of a significant troponin I increase associated with IORT [28]. Although we can conclude that IORT doesn’t seem to have an acute cardiotoxic effect, we would not, based on these finding, be able to claim that IORT has a beneficial effect on cardiac health, as there is no pathophysiological explanation for such a result.

Our study is limited by various factors simultaneously affecting cardiac toxicity, foremost the additional systemic therapy regimens consisting of chemotherapy or endocrine and/or targeted therapy and intraoperative narcotic management. Another limitation lies in a relatively small cohort size, despite being the largest trial currently addressing the aforementioned acute cardiac safety of IORT. The limited cohort size in turn causes a lack of statistical power to exclude small differences. Furthermore, the absence of a predefined statistical hypothesis comprises another limitation of our study. Also, the frequency of diabetes mellitus was not analyzed. Since this disease is well known to cause microvascular complications, it could be a confounder that we did not control for in our study. Finally, technical innovations such as DIBH can significantly lower cardiac dose exposure and decrease the risk of cardiac toxicity from EBRT [36, 37].

## Conclusion

In conclusion, these findings speak in favor of acute cardiac safety of IORT with low-energy x‑rays within the scope of BCS for early breast cancer. While IORT doesn’t seem to cause acute myocardial damage as inferred by troponin I and NT-proBNP dynamics, further follow-up studies are crucial to investigate the long-term cardiac effects of IORT.

## Caption Electronic Supplementary Material

Standardized NT-proBNP and Troponin I dynamics in the IORT group

Standardized NT-proBNP and Troponin I dynamics in the control group

Standardized NT-proBNP and Troponin I dynamics
