# Supplementary figures and images for: Cardiac serum marker alterations after intraoperative radiotherapy with low-energy x-rays in early breast cancer as an indicator of possible cardiac toxicity
Source: Strahlenther Onkol. 2020 Aug 19;197(1):39–47. doi: 10.1007/s00066-020-01671-3 (PMC7801302; doi:10.1007/s00066-020-01671-3)

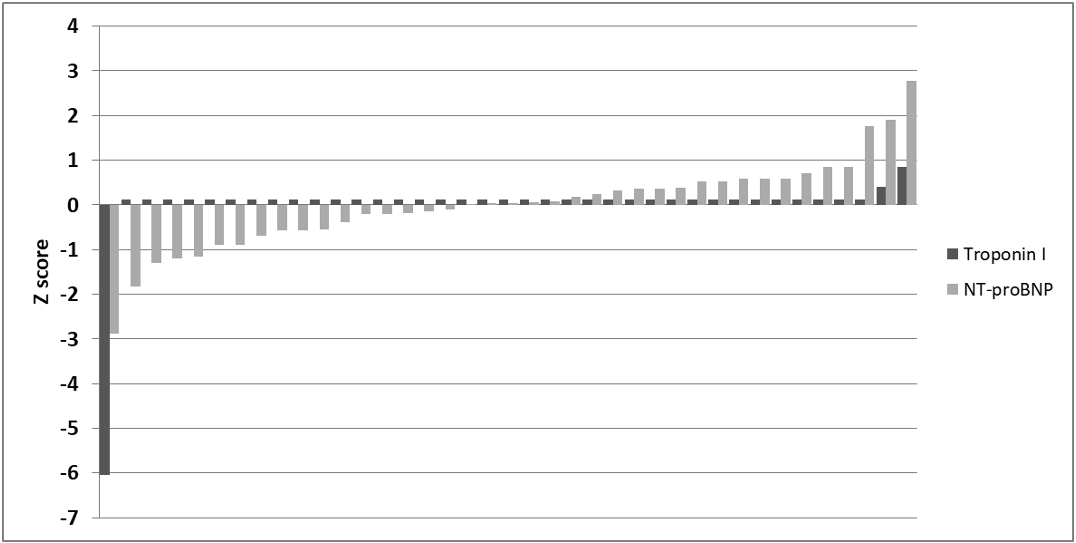

Supplement: Supplementary file 1 — Standardized NT-proBNP and Troponin I dynamics in the IORT group [file 66_2020_1671_MOESM1_ESM.png]

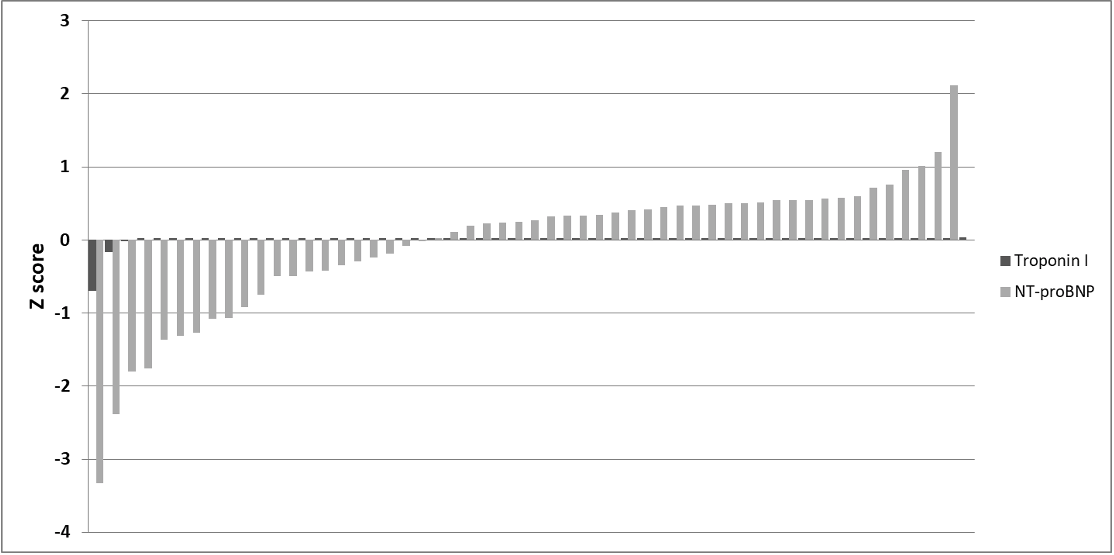

Supplement: Supplementary file 2 — Standardized NT-proBNP and Troponin I dynamics in the control group [file 66_2020_1671_MOESM2_ESM.png]

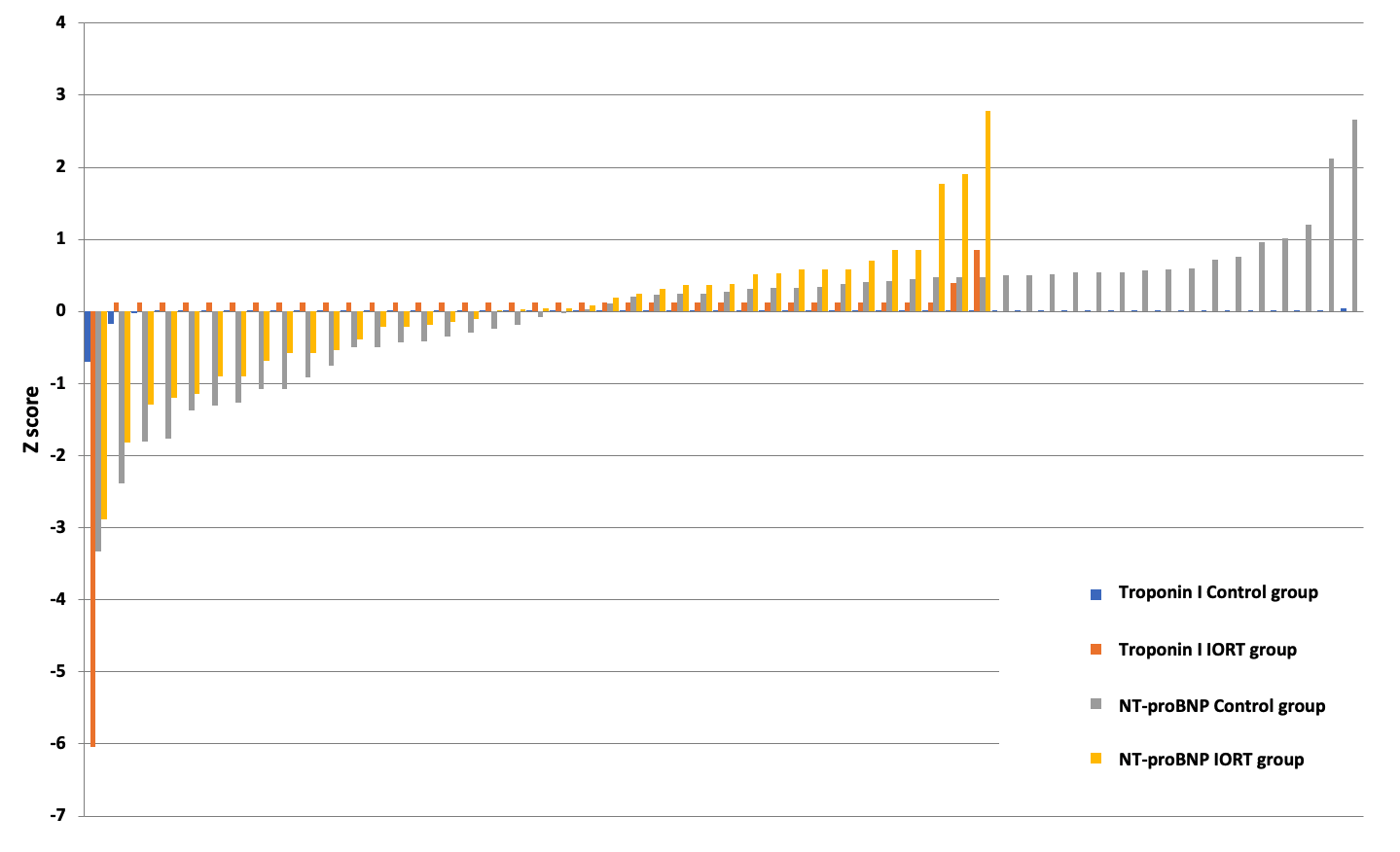

Supplement: Supplementary file 3 — Standardized NT-proBNP and Troponin I dynamics [file 66_2020_1671_MOESM3_ESM.png]
